# Influence of Antimicrobial Stewardship and Molecular Rapid Diagnostic Tests on Antimicrobial Prescribing for Extended-Spectrum Beta-Lactamase- and Carbapenemase-Producing Escherichia coli and Klebsiella pneumoniae in Bloodstream Infection

**DOI:** 10.1128/Spectrum.00464-21

**Published:** 2021-10-27

**Authors:** Ashlan J. Kunz Coyne, Anthony M. Casapao, Carmen Isache, James Morales, Yvette S. McCarter, Christopher A. Jankowski

**Affiliations:** a Department of Pharmacy, UF Health Jacksonville, Jacksonville, Florida, USA; b Department of Pharmacotherapy and Translational Research, UF College of Pharmacy, Jacksonville, Florida, USA; c Department of Medicine, UF Health Jacksonville, Jacksonville, Florida, USA; d Department of Pathology and Laboratory Medicine, UF Health Jacksonville, Jacksonville, Florida, USA; Hartford Hospital

**Keywords:** molecular rapid diagnostic test, antimicrobial stewardship program, extended-spectrum beta-lactamase-producing *Enterobacterales*, carbapenemase-producing *Enterobacterales*

## Abstract

The objective of this study was to evaluate whether the addition of the Verigene BC-GN molecular rapid diagnostic test to standard antimicrobial stewardship practices (mRDT + ASP) decreased the time to optimal and effective antimicrobial therapy for patients with extended-spectrum beta-lactamase (ESBL)- and carbapenemase-producing Escherichia coli and Klebsiella pneumoniae bloodstream infections (BSI) compared to conventional microbiological methods with ASP (CONV + ASP). This was a multicenter, retrospective cohort study evaluating the time to optimal antimicrobial therapy in 5 years of patients with E. coli or K. pneumoniae BSI determined to be ESBL- or carbapenemase-producing by mRDT and/or CONV. Of the 378 patients included (mRDT + ASP, *n* = 164; CONV + ASP, *n* = 214), 339 received optimal antimicrobial therapy (mRDT + ASP, *n* = 161; CONV + ASP, *n* = 178), and 360 (mRDT + ASP, *n* = 163; CONV + ASP, *n* = 197) received effective antimicrobial therapy. The mRDT + ASP demonstrated a statistically significant decrease in the time to optimal antimicrobial therapy (20.5 h [interquartile range (IQR), 17.0 to 42.2 h] versus 50.1 h [IQR, 27.6 to 77.9 h]; *P* < 0.001) and the time to effective antimicrobial therapy (15.9 h [IQR, 1.9 to 25.7 h] versus 28.0 h [IQR, 9.5 to 56.7 h]; *P* < 0.001) compared to CONV + ASP, respectively.

**IMPORTANCE** Our study supports the additional benefit of molecular rapid diagnostic test in combination with timely antimicrobial stewardship program (ASP) intervention on shortening the time to both optimal and effective antimicrobial therapy in patients with ESBL- or carbapenemase-producing Escherichia coli and Klebsiella pneumoniae bloodstream infections, compared to conventional microbiological methods and ASP. Gram-negative infections are associated with significant morbidity and mortality, often resulting in life-threatening organ dysfunction. Both resistance phenotypes confer resistance to many of our first-line antimicrobial agents with carbapenemase-producing *Enterobacterales* requiring novel beta-lactam and beta-lactamase inhibitor combinations or other susceptible non-beta-lactam antibiotics for treatment. National resistance trends in a cohort of hospitalized patients at U.S. hospitals during our study period demonstrate the increasing incidence of both resistance phenotypes, reinforcing the generalizability and timeliness of such analysis.

## INTRODUCTION

Gram-negative bloodstream infections (GN BSI) are associated with significant morbidity and mortality, often resulting in life-threatening organ dysfunction, such as sepsis and septic shock (1). This scenario has worsened, with Gram-negative *Enterobacterales* driving the emergence of drug-resistant pathogens, which are difficult to treat or even untreatable with conventional antimicrobials (2). Escherichia coli and Klebsiella pneumoniae are two members of the order *Enterobacterales* with a concerning increase in extended-spectrum beta-lactamases (ESBL) and carbapenemases. ESBL confers resistance to many of our first-line antimicrobial agents, including third-generation cephalosporins, penicillin-beta-lactamase inhibitor combinations, cefepime, and aztreonam (3). Patients with infections caused by ESBL-producing bacteria often require antimicrobial therapy within the carbapenem class of antimicrobial agents (ertapenem, meropenem, etc.), as they have been shown to have better outcomes than other beta-lactam therapies (4, 5). In contrast, carbapenemase-producing *Enterobacterales* demonstrate resistance to carbapenems and typically require novel beta-lactam and beta-lactamase combination antimicrobials (ceftazidime-avibactam, meropenem-vaborbactam, etc.) or other susceptible non-beta-lactam antibiotics for treatment (6). The two most common genes associated with ESBL and carbapenemases in these organisms are cefotaximase-Munich (CTX-M) and K. pneumoniae carbapenemase (KPC), respectively (7). For dynamic and costly infections such as BSI, timely and effective antimicrobial therapy is crucial to enhance patient survivability (8).

Molecular rapid diagnostic tests (mRDT) may help expedite identification of the causative pathogen (9). This produces the potential to administer immediate targeted antimicrobial therapy and lessen the clinical burdens related to ESBL- and carbapenemase-producing bacteria (10). A myriad of comprehensive, panel-based molecular diagnostic assays that detect common bloodstream pathogens and select antimicrobial resistance genes are now available for direct testing of positive blood cultures. The Verigene BC-GN system bloodstream infection test (Luminex Corporation, Northbrook, IL, USA) is a multiplex microarray platform that can detect organisms to the genus level for four genera (Acinetobacter spp., *Citrobacter* spp., Proteus spp., and Enterobacter spp.), four organisms to the species level (Escherichia coli, Klebsiella pneumoniae, Klebsiella oxytoca, and Pseudomonas aeruginosa), and six beta-lactamase genes (ESBL: CTX-M; carbapenemases: KPC, VIM, IMP, NDM, and OXA). Identification occurs within 2.5 h of Gram stain, compared to 30 or more hours with conventional microbiological methods, with a sensitivity and specificity of 97.1% and 99.5%, respectively (11, 12).

Antimicrobial stewardship programs (ASP) are interprofessional coordinated programs that implement strategies for appropriate antimicrobial use to optimize infection-related outcomes, while minimizing the unintended consequences of treating infections (i.e., the emergence of antimicrobial resistance or adverse drug reactions) (13, 14). In 2017, the Joint Commission recommended the implementation of ASP at all acute and critical care hospitals in the United States (15). The greatest impact of mRDT appears to occur when the tests are implemented in combination with ASP intervention to ensure that the test result is acted on in a timely manner (12, 16). The Infectious Diseases Society of America (IDSA) antimicrobial stewardship program guidelines advocate for the use of mRDT with ASP support and intervention as an addition to conventional methods for blood specimens to improve clinical outcomes (13). While previous studies have investigated the impact of utilizing mRDT and ASP intervention in patients with GN BSI, there are limited data on the use of mRDT for ESBL- and carbapenemase-producing *Enterobacterales* (12, 17). The purpose of this study was to evaluate whether the addition of mRDT to standard ASP practices (mRDT + ASP) decreased the time to optimal antimicrobial therapy for patients with either ESBL- or carbapenemase-producing E. coli and K. pneumoniae BSI compared to conventional microbiological methods with ASP (CONV + ASP).

## RESULTS

A total of 378 unique patients were included for evaluation. The most common reasons for study exclusion were having polymicrobial BSI (31%), having received a bone marrow and/or solid organ transplant (20%), and having been transferred in from an outside hospital with a known positive blood culture (19%). The mRDT + ASP (*n* = 164) and CONV + ASP (*n* = 214) were balanced with respect to the baseline characteristics (Table 1). The incidences of ESBL (91.5% versus 94.9%; *P* = 0.807) and carbapenemase-producing (8.5% versus 5.1%; *P* = 0.219) E. coli and K. pneumoniae in BSI were similar between mRDT + ASP and CONV + ASP, respectively. In the mRDT + ASP cohort, all resistance markers identified on the BC-GN test displayed phenotypic resistance on confirmatory Vitek 2 testing. The most common BSI sources for both mRDT + ASP and CONV + ASP were genitourinary (51.8% versus 45.8%) and intra-abdominal (12.2% versus 14.1%). Repeat blood cultures were collected in 148 of mRDT + ASP patients and 187 of CONV + ASP patients (90.2% versus 87.4%; *P* = 0.754). Infectious diseases (ID) consults were significantly more frequent for CONV + ASP compared to mRDT + ASP (82.2% versus 34.8%, respectively; *P* < 0.001).

**TABLE 1:** Comparison of baseline characteristics for the mRDT + ASP and CONV + ASP groups^*a*^

Characteristic^*b*^	mRDT + ASP group (*n *= 164)	CONV + ASP group (*n* = 214)	*P* value
Age (yrs) (mean ± SD)	59.5 ± 15.7	62.9 ± 16.9	0.054
Female sex	71 (43.3)	95 (44.4)	0.100
Race/ethnicity			
Black	79 (48.2)	58 (27)	0.189
Non-Hispanic white	76 (46.3)	143 (66.8)	0.096
Hispanic	9 (5.5)	9 (4.2)	0.254
Beta-lactam allergy	33 (20.1)	35 (16.4)	0.301
NH/LTC residence	36 (22)	41 (19.2)	0.562
Immunosuppressive medication	13 (7.9)	13 (6.1)	0.275
Surgical procedure within previous 30 days	34 (20.7)	41 (19.2)	0.103
Gram-negative infection within 6 months	79 (48.2)	87 (40.7)	0.139
History of infection due to ESBL-producing *Enterobacterales*	54 (32.9)	61 (28.5)	0.759
History of infection due to carbapenemase-producing *Enterobacterales*	6 (3.7)	2 (0.9)	0.084
Charlson comorbidity index (mean ± SD)	6.1 ± 3.4	5.5 ± 2.9	0.275
Pitt bacteremia score (mean ± SD)	3.1 ± 2.4	3.4 ± 2.7	0.439
Hospital-acquired infection	84 (51.2)	94 (43.9)	0.233
Index culture results			
ESBL-producing *Enterobacterales*	150 (91.5)	203 (94.9)	0.807
E. coli	86 (57.3)	129 (63.5)	0.237
K. pneumoniae	64 (42.7)	74 (36.5)	
Carbapenemase-producing *Enterobacterales*	14 (8.5)	11 (5.1)	0.219
E. coli	0 (0)	1 (9)	0.441
K. pneumoniae	14 (100)	10 (91)	
ID consult	57 (34.8)	176 (82.2)	<0.001
Sources			
Endovascular	13 (7.9)	22 (10.3)	
Intra-abdominal	20 (12.2)	30 (14.1)	
Genitourinary	85 (51.8)	98 (45.8)	
Respiratory	12 (7.3)	20 (9.3)	
SSTI	15 (9.1)	19 (8.9)	
Other/unknown	19 (11.6)	25 (11.7)	

a Data presented as *n* (%) unless specified otherwise.

b NH/LTC, nursing home/long-term care; ESBL, extended-spectrum beta-lactamase; ID, infectious diseases; SSTI, skin and soft tissue infection.

**FIG 1:**
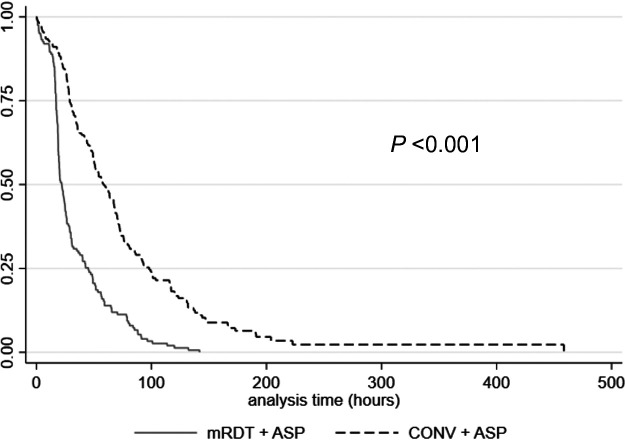
Kaplan-Meier time to optimal antimicrobial therapy estimates for the mRDT + ASP and CONV + ASP groups.

**FIG 2:**
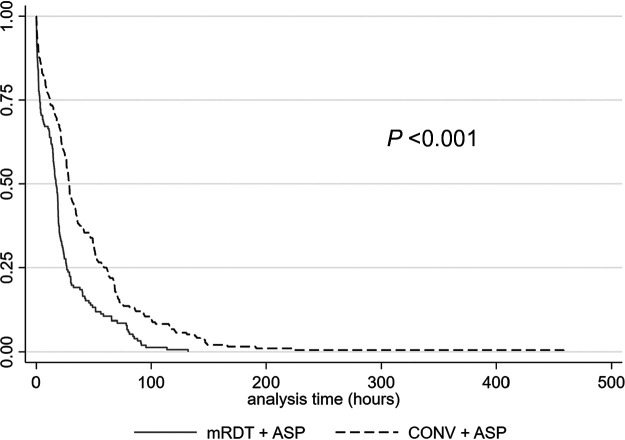
Kaplan-Meier time to effective antimicrobial therapy estimates for the mRDT + ASP and CONV + ASP groups.

Overall, 89.7% (339/378) received optimal antimicrobial therapy, which breaks down to 98.2% (161/164) of the mRDT + ASP group compared to 83.2% (178/214) of the CONV + ASP group (*P* < 0.001), demonstrating a statistically significant decrease in the time to optimal antimicrobial therapy (20.5 h [interquartile range (IQR),17.0 to 42.2 h] versus 50.1 h [IQR, 27.6 to 77.9 h], respectively; *P* < 0.001) (Fig. 1). In total, 95.2% (360/378) received effective antimicrobial therapy, with mRDT + ASP (*n* = 163/164) demonstrating a statistically significant decrease in time to effective antimicrobial therapy compared to CONV + ASP (*n* = 197/214) (15.9 h [IQR, 1.9 to 25.7 h] versus 28.0 h [IQR, 9.5 to 56.7 h], respectively; *P* < 0.001) (Fig. 2). Effective and optimal therapies for mRDT + ASP and CONV + ASP are listed in Table 2. According to the *post hoc* revised analysis of the time to optimal antimicrobial therapy, a total of 339/353 (96.0%) patients with ESBL-producing E. coli or K. pneumoniae BSI received effective therapy, with 335/339 (98.8%) of those patients receiving either a carbapenem or piperacillin-tazobactam as definitive therapy, and thus were included for analysis. In this *post hoc* analysis, mRDT + ASP (*n* = 150/150) again demonstrated a significant decrease in the revised time to optimal antimicrobial therapy compared to CONV + ASP (*n* = 185/189) (17.1 h [IQR, 1.8 to 26.4 h] versus 28.8 h [IQR, 11.3 to 62.3 h], respectively; *P* < 0.001).

**TABLE 2:** Effective and optimal antimicrobial therapy for the mRDT + ASP and CONV + ASP groups^*a*^

Antimicrobial therapy^*b*^	mRDT + ASP group (*n *= 164)	CONV + ASP group (*n *= 214)	*P* value
ESBL-producing E. coli or K. pneumoniae	150 (91.5)	203 (94.9)	0.807
Effective therapy	149 (99.3)	187 (92.1)	0.002
Amikacin	1 (0.7)	10 (5.3)	
Ciprofloxacin	1 (0.7)	1 (0.5)	
Colistin	0 (0)	1 (0.5)	
Ertapenem	26 (17.3)	30 (29.9)	
Gentamicin	2 (1.3)	1 (0.5)	
Levofloxacin	2 (1.3)	4 (2.1)	
Meropenem	61 (40.7)	84 (44.9)	
Piperacillin-tazobactam	56 (37.3)	56 (29.9)	
Tobramycin	1 (0.67)	0 (0)	
Optimal therapy	148 (98.7)	168 (82.8)	<0.001
Ertapenem	66 (44.6)	35 (23.6)	
Meropenem	82 (55.4)	133 (89.9)	
Carbapenemase-producing E. coli or K. pneumoniae	14 (8.5)	11 (5.1)	0.219
Effective therapy	13 (92.9)	10 (90.9)	0.999
Amikacin	1 (7.7)	5 (0.5)	
Ceftazidime-avibactam	2 (15.4)	2 (0.2)	
Ceftolozane-tazobactam	0 (0)	1 (0.1)	
Colistin	1 (7.7)	1 (0.1)	
Levofloxacin	1 (7.7)	0 (0)	
Polymyxin	3 (23.1)	0 (0)	
Sulfamethoxazole-trimethoprim	1 (7.7)	1 (0.1)	
Tigecycline	4 (30.8)	0 (0)	
Optimal therapy	13 (92.9)	10 (90.9)	0.999
Amikacin	1 (7.7)	3 (0.3)	
Ceftazidime-avibactam	3 (23.1)	4 (0.4)	
Ceftolozane-tazobactam	0 (0)	1 (0.1)	
Colistin	1 (7.7)	1 (0.1)	
Levofloxacin	1 (7.7)	0 (0)	
Polymyxin	3 (23.1)	0 (0)	
Sulfamethoxazole-trimethoprim	0 (0)	1 (0.1)	
Tigecycline	4 (30.8)	0 (0)	

a Data presented as *n* (%) unless specified otherwise.

b ESBL, extended-spectrum beta-lactamase.

**FIG 3:**
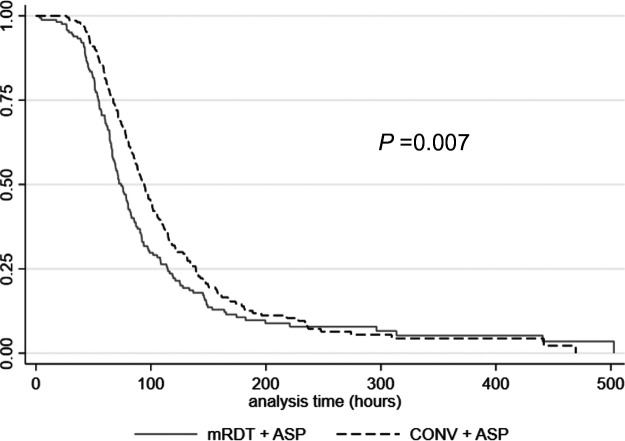
Kaplan-Meier time to microbial clearance estimates for the mRDT + ASP and CONV + ASP groups.

In addition, the time to microbial clearance was significantly lower for mRDT + ASP compared to CONV + ASP (71.9 h [IQR, 54.1 to 108.5 h] versus 91.2 h [IQR, 64.6 to 134.3 h]; *P* = 0.007), respectively (Fig. 3). The mRDT + ASP group demonstrated a 5.6% decrease in all-cause hospital mortality compared to CONV + ASP (8.0% versus 13.6%; *P* = 0.088).

The length of stay (LOS) was similar between mRDT + ASP and CONV + ASP (12.5 days [IQR, 7 to 29 days] versus 12 days [IQR, 7 to 23 days]; *P* = 0.465), with the mRDT + ASP group demonstrating a significant decrease in infection-related LOS (3 days [IQR, 2 to 4 days] versus 4 days [IQR, 3 to 5 days], respectively; *P* < 0.001). No significant differences were detected in the mRDT versus CONV groups for the 30-day (27.4% versus 20.1%; *P* = 0.094), 60-day (36.6% versus 28.0%; *P* = 0.093), or 90-day (38.4% versus 31.8%; *P* = 0.179) readmission rates or the Clostridioides difficile rates (6.1% versus 3.3%; *P* = 0.189), respectively. No overall difference in hospital charges ($114,649.59 versus $88,218.40; *P* = 0.711) and infection-related charges ($43,488.94 versus $39,695.20; *P* = 0.960) was identified between mRDT + ASP and CONV + ASP, respectively.

## DISCUSSION

Our study supports the additional benefit of mRDT to ASP intervention on shortening the time to optimal antimicrobial therapy with ESBL- or carbapenemase-producing E. coli and K. pneumoniae BSI compared to CONV + ASP intervention. Two quasi-experimental studies conducted by Bork et al. and Sothoron et al. demonstrated that coupling Verigene BC-GN mRDT to ASP decreased the time to optimal therapy by 18.3 and 10.8 h, respectively, compared to CONV + ASP for GN BSI (12, 17). Our findings align with previous literature describing the additional benefit of utilizing a mRDT for the identification of pathogens from a blood culture. However, these studies were limited by their small sample size and low rates of resistance genes isolated, making the data difficult to generalize to other institutions or resistant bacteria. A strength of our study is the inclusion of patients only if they had culture-positive ESBL- or carbapenemase-producing E. coli and K. pneumoniae BSI. National resistance trends in a cohort of hospitalized patients at 890 U.S. hospitals during our study period demonstrated the increasing incidence of both resistance phenotypes, reinforcing the generalizability and timeliness of such analysis (18). Based on previous studies comparing the time to optimal antimicrobial therapy between mRDT + ASP and CONV + ASP in patients with GN BSI, our study includes one of the largest sample sizes of patients with ESBL- or carbapenemase-producing E. coli and K. pneumoniae BSI to date. Another strength of our study is the similarity in the median time to optimal therapy for Gram-negative BSI at the mRDT institution prior to mRDT implementation (49.3 h) compared to that demonstrated in the CONV + ASP group in this study (50.1 h). These findings demonstrate that while minor practice variation between sites may occur, the coordination of mRDT + ASP results in a faster time to optimal therapy compared to either mRDT or ASP alone (12, 16). One of the keystone principles of all effective ASP is to ensure the “right drug for the right patient.” ASPs often review historical patient-specific microbiology data to aide in empirical antimicrobial selection. In our multicenter study, only one-third of the patients had a history of an ESBL- or carbapenemase-producing infection. Therefore, an ASP review of the culture history alone would not have been sufficient to predict these resistant bacterial infections without the aid of mRDT for the majority of the cases. Other studies have demonstrated that patients presenting with ESBL- and carbapenemase-producing bacteria in BSI are less likely to be started on appropriate empirical antimicrobial therapy (19, 20). Additionally, while antimicrobial de-escalation is a pillar of ASP, ensuring that patients receive timely and effective antimicrobial therapy, even if it requires therapeutic escalation, is of equal importance to ensure optimal patient care. Our study reinforces the positive impact that mRDT added to ASP practice can have on the timely initiation of optimal antimicrobial therapy in patients with ESBL- or carbapenemase-producing *Enterobacterales* in BSI. In addition, the more rapid time to optimal therapy in the mRDT + ASP group may explain the improved time to microbial clearance in this group, but more studies are warranted to validate this finding.

There were some limitations to this study. The data were retrospectively extracted from the electronic medical record (EMR) in a nonblinded manner, which allowed for potential information bias since it is unknown whether unmeasured or unreported confounders might have affected the clinical outcomes. Practice site variations, including but not limited to differences in patient demographics, medical practices, and variability in hospital charges for similar services, may exist between the two academic medical centers, although the study design excluded potential large enrollment differences in transplant and oncology patients due to known specific service imbalances and the severity of comorbidities between groups. Although repeat blood culture rates and LOS were similar between the groups, other variations in the workflow of ordering and collecting repeat blood cultures or discharge planning may have also been present and affected our secondary outcomes of time to microbial clearance and infection-related LOS. The rate of infectious diseases consults was also different between groups, in which the CONV + ASP had a significantly higher rate of consultations compared to mRDT + ASP, even though the time to optimal antimicrobial therapy was significantly shorter in the mRDT + ASP group. While neither mRDT + ASP nor CONV + ASP required infectious diseases consults for positive blood cultures, both groups required infectious diseases consults to order the novel beta-lactam/beta-lactamase inhibitor combinations (e.g., ceftazidime-avibactam) included as effective and optimal therapy in this study. Additionally, CONV + ASP required carbapenem orders be approved by an infectious diseases (ID) pharmacist, postgraduate year two (PGY2) ID pharmacy resident, or another member of the infectious diseases consult service member. If a new order for a carbapenem was placed outside normal business hours (0800 to 1700), the ordering prescriber was able to select “after hours” as the order approver. If “after hours” was selected, order approval was still required from an ID pharmacist, PGY2 ID pharmacy resident, or another infectious diseases consult service member the following day.

Additionally, piperacillin-tazobactam was excluded as an optimal antimicrobial therapy for ESBL-producing bacteria in BSI. This exclusion was based on data from the MERINO trial, in which piperacillin-tazobactam did not demonstrate noninferiority to carbapenem therapy for ESBL-producing bacteria in BSI (5). When accounting for ESBL-producing isolates determined to be susceptible to piperacillin-tazobactam using Vitek, E test, and broth microdilution assays per CLSI or EUCAST breakpoints, patients in the MERINO trial who received piperacillin-tazobactam as definitive therapy for ESBL-producing bacteria in BSI demonstrated a higher mortality of 8.7%, compared to 3.7% in those receiving meropenem as definitive therapy. This finding reinforces our exclusion of piperacillin-tazobactam as optimal therapy for patients with ESBL-producing bacteria in BSI. Our study includes patients hospitalized prior to the publication of the MERINO trial, and during that time there was a professional viewpoint that piperacillin-tazobactam could be an alternative effective agent to carbapenems for ESBL-producing bacteria in BSIs; as a result, there is the limitation of temporality in our study’s primary end point. To address this limitation, we conducted a *post hoc* analysis of the time to optimal therapy in patients with ESBL-producing bacteria in BSI receiving either piperacillin-tazobactam or a carbapenem as definitive therapy. Even with the inclusion of piperacillin-tazobactam as optimal therapy, mRDT + ASP demonstrated a significantly shorter time to optimal therapy compared to CONV + ASP. Another recognized limitation of our study is the exclusion of polymicrobial infections, which excluded approximately 20% of the initial samples screened. Accurate identification of polymicrobial samples is a known limitation of the Verigene BC-GN rapid test (21).

## CONCLUSION

Despite these limitations, our study demonstrates that mRDT + ASP, compared to CONV + ASP, may shorten the time to both optimal and effective antimicrobial therapy, as well as time to microbial clearance, in patients with ESBL- or carbapenemase-producing E. coli and K. pneumoniae BSI. Antimicrobial stewardship programs can use these data to help justify the need for mRDT to quickly identify patients and promote optimal antimicrobial therapy in patients with ESBL- or carbapenemase-producing E. coli and K. pneumoniae BSI. Future studies should assess whether the shorter time to microbial clearance translates to a shorter necessary duration of therapy, and a comparison of outcomes between the specific carbapenem agents utilized as optimal therapy for ESBL-producing bacteria in BSI to further elucidate the full impact of this intervention on clinical and economic outcomes.

## MATERIALS AND METHODS

This was a retrospective cohort study conducted at two academic medical centers, UF Health Jacksonville in Jacksonville, FL (UFHJ), and UF Health Shands in Gainesville, FL (UF Shands). Throughout the study period, UFHJ identified patient blood cultures using Verigene BC-GN in addition to concomitant conventional microbiological methodologies and ASP intervention (mRDT + ASP), while UF Shands utilized conventional microbiological methods with ASP intervention (CONV + ASP). The institutional ASP operating at both medical centers consisted of infectious diseases pharmacists and physicians. Adults age 18 years or older admitted from February 2014 through July 2019 with blood cultures positive for E. coli or K. pneumoniae available in the EMR were evaluated for study inclusion. Only the first positive blood culture for each patient determined to be ESBL- or carbapenemase-producing was included during the entire study period.

Patients were excluded if they had a polymicrobial BSI, had transferred in from an outside facility with a previously identified positive blood culture, were bone marrow/solid organ transplant recipients, had a diagnosis of cancer/febrile neutropenia, were incarcerated, were enrolled in a concomitant research study, or died before the culture results. In the mRDT + ASP group, blood samples were directly inoculated with patient blood samples and incubated in the Bactec 9240 (2014 to 2015) and Bactec FX (2015 to 2019) systems. If bacterial growth was detected, Gram staining was performed, and the results were called to the unit nurse and/or provider as critical results. Verigene BC-GN testing was performed on the first positive blood culture according to the manufacturer’s specifications (11). Microbiology paged the ASP and other trained pharmacists, 24 h per day, 7 days per week, with BC-GN test results, which were also called to the unit nurse and/or primary team as critical results. Clinical algorithms for mRDT results were available to all pharmacists during the study period. During nonstandard ASP hours (1700 to 0800), the clinical response to BC-GN alerts was at the discretion of the pharmacist on duty, as there was no standardized protocol for pharmacist response to Verigene BC-GN alerts. Any BC-GN result reported during nonstandard ASP hours was reviewed during business hours (0800 to 1700) by the ASP and other trained pharmacists 7 days per week for potential optimization. All BC-GN test results were confirmed by conventional microbiological methods as part of standard practice. Susceptibility and confirmatory testing were conducted using the Vitek 2 system for ESBL-producing bacteria. The modified Hodge test (2014 to 2017) and meropenem E test using new breakpoints (2017 to 2019) were used for susceptibility and confirmatory testing of carbapenemase-producing bacteria (22, 23).

In the CONV + ASP group, blood samples were directly inoculated with patient blood samples and incubated in the Bactec 9240 (2014 to 2016) and Bactec FX (2016 to 2019) systems. If bacterial growth was detected, Gram staining was performed, and the results were called to the unit nurse and primary team as critical results. The blood culture broth was inoculated on solid medium, with growth identified using the Vitek 2 system, which was also utilized for antimicrobial susceptibility and confirmatory testing of ESBL-producing bacteria. For carbapenemase-producing isolates, the modified Hodge test (2014 to 2017) and Xpert Carba-R assay (2018 to 2019) were used (bioMérieux, Durham, NC) (24). All first-time positive blood cultures containing ESBL- or carbapenemase-producing bacteria were reported to the unit nurse and/or primary team as critical results. Antimicrobial stewardship and other trained pharmacists then reviewed the prescribed antimicrobial agent(s) and provided pharmacotherapeutic recommendations to prescribers as microbiology information became available. Both groups utilized Clinical and Laboratory Standards Institute (CLSI) breakpoints for interpretation of the susceptibilities for all causative organisms and had active ASP throughout the study period (25).

Data were collected retrospectively from the EMR and included baseline, antimicrobial administration, microbiological, clinical, and outcome data, as well as hospital charges. The comorbidity burden was estimated using the Charlson comorbidity index and the baseline severity of BSI using the Pitt bacteremia score (19, 26). BSI sources were defined according to the Centers for Disease Control and Prevention (CDC) criteria (27). Dual evaluation of the collected data pertaining to the primary outcome was completed by an infectious diseases physician and/or pharmacist in a manner blinded to the medical center.

The primary outcome was time to optimal antimicrobial therapy, defined as the time elapsed between index blood culture collection to the first administration of carbapenem therapy for ESBL-producing bacteria or either ceftazidime-avibactam, meropenem-vaborbactam, or at least one drug active *in vitro* (e.g., fluoroquinolones, sulfamethoxazole-trimethoprim, tigecycline, aminoglycosides, or polymyxins) for carbapenemase-producing bacteria (28, 29).

Secondary outcomes for both medical centers were time to effective antimicrobial therapy defined as the time elapsed between index blood culture collection to first administration of antimicrobial therapy with *in-vitro* susceptibility (e.g. ceftazidime-avibactam, meropenem-vaborbactam, fluoroquinolones, sulfamethoxazole-trimethoprim, tigecycline, aminoglycosides or polymyxins for carbapenemase-producing bacteria and piperacillin-tazobactam or any of the aforementioned agents for ESBL-producing bacteria). For patients with infections due to ESBL-producing *E. coli* or *K. pneumoniae* started empirically and continued on carbapenem therapy, or those with carbapenem-resistant *E. coli* or *K. pneumoniae* started empirically on combination therapy that was active *in-vitro*, time to effective therapy and time to optimal therapy were considered interchangeable. Time to microbial clearance defined as the time elapsed between index blood culture collection to a collection of first negative blood culture in patients with at least one repeat blood culture collected, all-cause in-hospital mortality, length of stay (LOS), and infection-related LOS defined as the time elapsed between index blood culture collection to first negative repeat blood culture or hospital discharge, whichever came first. Repeat blood cultures were obtained as a result of routine clinical practice and by the discretion of the treatment team. Other secondary outcomes included: 30-, 60- and 90-day readmission rates, *Clostridioides difficile* rates, and hospital charges and infection-related charges defined as patient-specific hospital charges accrued throughout the entire hospitalization and from index blood culture collection to completion of antimicrobial therapy or discharge for the BSI in US dollars, respectively.

In addition, a *post hoc* revised time to optimal antimicrobial therapy analysis was performed in patients with ESBL-producing E. coli or K. pneumoniae BSI initiated on effective therapy with receipt of either a carbapenem or piperacillin-tazobactam as definitive therapy to help control for potential prescribing variances concerning the professional interpretation of beta-lactamase inhibitor combinations for the treatment of susceptible ESBL-producing *Enterobacterales* during the study period.

Descriptive statistics were performed between mRDT + ASP and CONV + ASP. The Kaplan-Meier method was used with the log-rank test to analyze the primary outcome of time to optimal antimicrobial therapy, secondary outcomes of both time to effective therapy and time to microbial clearance, and the *post hoc* revised analysis of time to optimal antimicrobial therapy. Bivariate analysis was performed using the chi-square test/Fisher’s exact test and Student’s *t* test or the Wilcoxon rank sum test for continuous variables, as appropriate. A *P* value of less than 0.05 was considered statistically significant. All analyses were performed using IBM SPSS Statistics for Windows version 26 (Armonk, NY, USA).
